# Development of electrocardiogram intervals during growth of FVB/N neonate mice

**DOI:** 10.1186/1472-6793-10-16

**Published:** 2010-08-24

**Authors:** Christopher R Heier, Thomas G Hampton, Deli Wang, Christine J DiDonato

**Affiliations:** 1 Human Molecular Genetics Program, Children's Memorial Research Center, 2300 Children's Plaza, PO Box 211, Chicago, IL 60614, USA; 2 Department of Pediatrics, Feinberg School of Medicine, Northwestern University, Chicago, IL, 60611, USA; 3 Mouse Specifics, Inc., Boston, MA, 02169, USA; 4 Biostatistics Research Core, Children's Memorial Research Center, Feinberg School of Medicine, Northwestern University, Chicago, IL, 60611, USA

## Abstract

**Background:**

Electrocardiography remains the best diagnostic tool and therapeutic biomarker for a spectrum of pediatric diseases involving cardiac or autonomic nervous system defects. As genetic links to these disorders are established and transgenic mouse models produced in efforts to understand and treat them, there is a surprising lack of information on electrocardiograms (ECGs) and ECG abnormalities in neonate mice. This is likely due to the trauma and anaesthesia required of many legacy approaches to ECG recording in mice, exacerbated by the fragility of many mutant neonates. Here, we use a non-invasive system to characterize development of the heart rate and electrocardiogram throughout the growth of conscious neonate FVB/N mice.

**Results:**

We examine ECG waveforms as early as two days after birth. At this point males and females demonstrate comparable heart rates that are 50% lower than adult mice. Neonatal mice exhibit very low heart rate variability. Within 12 days of birth PR, QRS and QTc interval durations are near adult values while heart rate continues to increase until weaning. Upon weaning FVB/N females quickly develop slower heart rates than males, though PR intervals are comparable between sexes until a later age. This suggests separate developmental events may contribute to these gender differences in electrocardiography.

**Conclusions:**

We provide insight with a new level of detail to the natural course of heart rate establishment in neonate mice. ECG can now be conveniently and repeatedly used in neonatal mice. This should serve to be of broad utility, facilitating further investigations into development of a diverse group of diseases and therapeutics in preclinical mouse studies.

## Background

Congenital heart disease and autonomic nervous system (ANS) dysfunction represent common and potentially deadly problems in the pediatric clinic [1-3]. Electrocardiography (ECG) is the best diagnostic tool and biomarker available for cardiac and ANS defects found in a spectrum of pediatric disorders. Accordingly, newborns and infants are routinely monitored via ECG and there is a wealth of human data available. Through early detection and intervention lives are now being extended by early diagnosis and treatment of arrhythmia associated diseases such as Holt-Oram syndrome, Lupus, familial dysautonomia, and DiGeorge syndrome. Links in ECG abnormalities are also being established to such previously mysterious diseases as sudden infant death syndrome (SIDS) and sudden cardiac arrest [4-6].

Current techniques in molecular genetics allow us to focus on the precise molecular basis for arrhythmias and are revealing a wide array of causes. Channelopathies [7,8], autonomic abnormalities, metabolic deficits, myocardial defects [9,10], drug toxicity, autoimmune disease, and neuromuscular disease [11,12] can all manifest arrhythmia. Now that many of the genes associated with such disorders have been identified, mouse models are being produced to study the mutations that result in them. Studies of Brugada syndrome, modelled in *Scn5a *mutant mice, have been instrumental in understanding channelopathies [13]. In neuromuscular disease *mdx *mice reproduce cardiac complications and arrhythmias found in Duchenne muscular dystrophy, including ANS dysfunction [14,15]. Studies in the *nmd *mouse model revealed a previously unobserved cardiomyopathy and arrhythmia later uncovered in spinal muscular atrophy with respiratory distress (SMARD) patients with extended survival [16]. Conduction defects such as ventricular pre-excitation displayed in Wolff-Parkinson-White syndrome are faithfully reproduced in *PRKAG2 *and *Alk3 *transgenic mice [17,18]. Finally, mutant mice that reproduce AV block and congenital heart defects were vital in elucidating a molecular pathway, encompassing Nkx2.5, Tbx5 and Id2, that is essential for development of the cardiac conduction system and is compromised in Holt-Oram syndrome [19-21].

In addition to elucidating the cause of ANS and cardiovascular disorders, transgenic mouse models are vital in developing therapeutics for them [22-24]. Surprisingly, there is little information on the development of ECG patterns within such animal models. The reason for this is likely due to the invasiveness, restraint, and/or surgery associated with many previous methods for obtaining ECG readings. Such methods may simultaneously interfere with the heart rate itself or jeopardize the life of groups of mice that are already very fragile [25-29]. Here, we utilize a passive and non-invasive system to characterize the development of various ECG parameters in growing mice [30]. The passive nature eliminates interference of anesthesia, drugs, and human handling on the heart beat of fragile groups of mice still undergoing autonomic maturation. It also allows for ECG acquisition in populations for which telemetry is not applicable due to age/size limitations, and for which experimenters wish not to sacrifice valuable mutant subjects for *ex vivo *analyses. The application of an acclimation period, in addition to the use of an externally heated warming stage to maintain nesting/body temperature, help to most closely ensure resting physiological states. Our study and data in a widely utilized inbred mouse strain should translate to a broad range of studies involving cardiac disorders, complications, and therapeutic efficacy in mouse models of human pediatric diseases.

## Results

**Figure 1:**
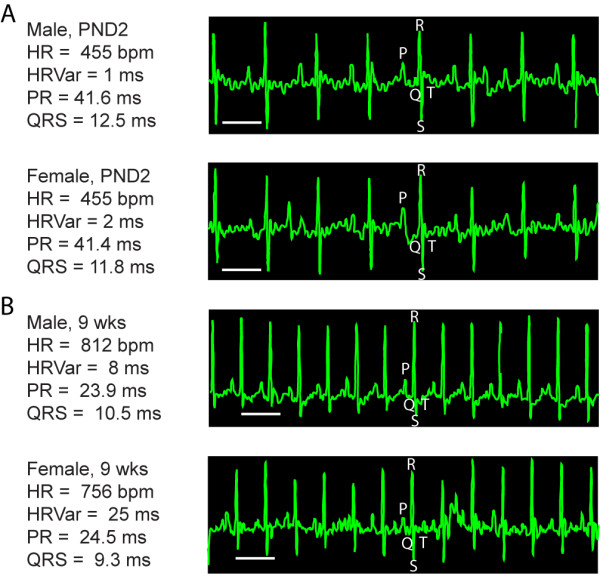
**ECG recordings at neonate and adult time points**. A) Tracing of representative ECG recordings from PND2 neonate FVB/N mice. All graphs show some tremor of the isoelectric line due to the mouse being conscious and unrestrained. B) Tracing of representative adult mouse ECG readings. Note the development of a slower adult heart rate in female (bottom) as compared to male (top) mice. P, Q, R, S, and T waveforms are denoted by their individual letters. Scale Bar = 100 ms.

We non-invasively recorded resting ECGs longitudinally in FVB/N neonates every two days from postnatal day (PND) 2 or 4 through PND14. We also recorded ECGs in young mice on the days immediately preceding and following weaning (weaning performed on PND21), then again at 4 weeks and 9 weeks of age. All mice were conscious, unsedated and unrestrained. Statistical analyses were performed with the aim of analyzing the relationship of gender differences and age over the development of ECG waveform intervals. Representative ECG tracings are depicted in Figure 1.

### Differential development of ECG interval durations in male and female mice

**Figure 2:**
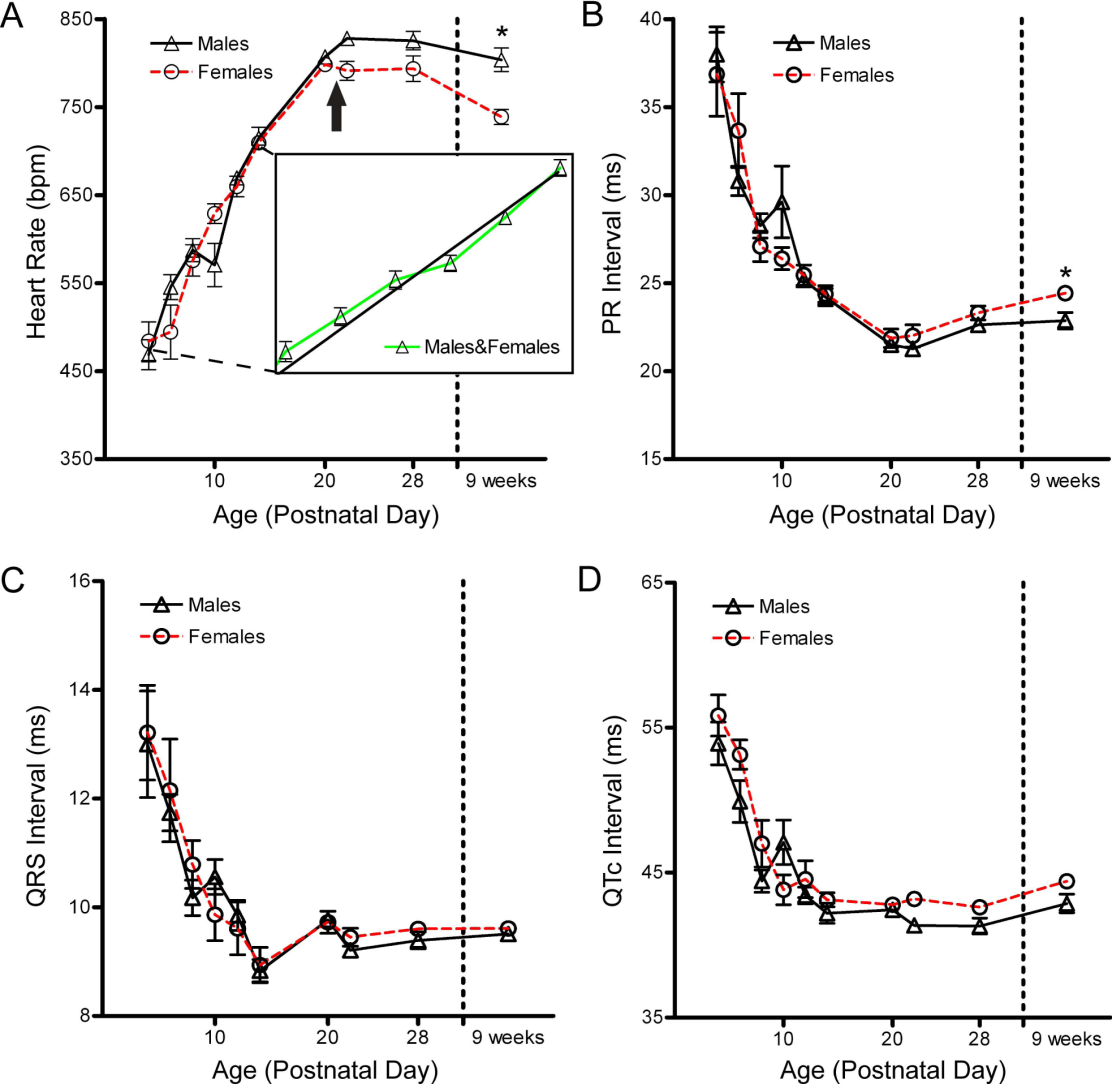
**Development of ECG interval durations in maturing FVB/N neonates**. Electrocardiograms were recorded for twenty mice assayed longitudinally from PND4 to 9 weeks of age. A) Heart rate of male and female mice throughout development. A strong interaction effect (*P *≤ 0.001) between age and sex is present during development. (*P ≤ 0.05, linear mixed model, n = 8 females and 12 males). Arrow denotes point at which mice were weaned. Inset in (A) illustrates the linear nature of heart rate development in young neonates, with best fit linear regression of combined male and female heart rate data from seventeen neonates collected longitudinally every day from PND2 to PND14. The average slope of individuals is 22 ± 4 bpm per day, with R^2 ^= 0.85 ± 0.11. B) PR interval during development. No interaction effect is present over development, however isolated analysis of the 9 week time point confirms a sex based difference is present in adults. (*P ≤ 0.05, student's *t*-test) C) QRS interval, and D) corrected QT interval (QTc) are presented as mean ± SEM, with no significant differences or interaction effects present.

We detected no difference between sexes in heart rate at any neonatal time point. Comparable heart rates were maintained through PND20 (females 798 ± 12 bpm versus males 807 ± 12 bpm). However immediately after weaning, at PND22, heart rates became differentiated between sexes (Figure 2A). The heart rate of females showed a small decrease from PND20 to PND22, while the heart rate of males continued to increase, with two sexes reaching values of 791 ± 31 bpm and 828 ± 22 bpm, respectively (Figure 2A). This resulted in a strong interaction effect (*P *≤ 0.001) between gender and age over development. A significant gender difference was absent in neonates but present in adult mice, with 739 ± 25 bpm in females and 804 ± 47 bpm in males (*P *≤ 0.05, linear mixed model with the randomized intercept, Bonferroni post-hoc corrected p-value)

An interaction effect between gender and age was not observed over development for PR interval, QRS interval, or QTc interval (Figures 2B-D). Strong effects of age were detected on all ECG parameters (*P *≤ 0.0001), while analysis of waveforms did not resolve a gender based difference over development for these three parameters. Through four weeks of age, comparable values were observed in females and males for PR intervals (23.3 ± 1.1 ms and 22.6 ± 1.3 ms, respectively), QRS intervals (9.6 ± 0.3 ms and 9.4 ± 0.5 ms), and QTc intervals (42.6 ± 1.2 ms versus 41.3 ± 1.9 ms) (Figures 2B-D). Isolated analyses comparing male and female data at 9 weeks however confirmed previously published results of gender based differences in PR intervals of adult mice [31]. Females showed an elongation of PR interval compared to males at this time point (24.4 ± 0.8 ms versus 22.9 ± 1.6 ms, *P *≤ 0.05, Student's *t*-test). No differences were observed between genders for QRS interval or QTc interval, though QTc intervals did exhibit a trend of slightly higher values in females, again in agreement with established data. These data indicate that the slower heart rate in young adult females at this age is not caused by differences in atrioventricular conduction or a delay between atrial and ventricular depolarization events.

### Development of ECG waveform intervals in neonate FVB/N mice

Since no differences were observed in any parameter between sexes prior to weaning, we pooled the data to examine the development of ECG interval durations in neonates (Table 1). Average heart rates were observed to rise steadily in developing neonates at a rate of approximately 25 bpm per day, from ~425 bpm at PND2 to ~725 bpm on PND14. To determine the linearity of heart rate development during neonate ages, linear regression was performed on longitudinal data from seventeen individual mice assayed at every neonatal time point from PND2 to PND14. Individual neonates exhibited linear heart rate development characterized by an average slope of 22 ± 4 bpm per day from with a mean R^2 ^of 0.85 ± 0.11 (Figure 2A). Heart rates continued to rise until PND20-22, where they reached peak values before a slow decline through 9 weeks of age. Such a slow decline was supportive of data from previous studies in aging mice [32].

**Table 1:** ECG indices in developing neonatal mice

Parameter	PND2	PND4	PND6	PND8	PND10	PND12	PND14
n =	17	20	20	20	20	20	20
HR (bpm)	424 ± 62	475 ± 59	525 ± 69	583 ± 46	594 ± 74	666 ± 25	713 ± 35
HRVar (ms)	3.2 ± 2.6	4.2 ± 4.3	12.4 ± 14.4	11.3 ± 10.5	16.5 ± 13.6	19.9 ± 10.5	18.0 ± 12.9
PR (ms)	45.3 ± 7.9	37.5 ± 5.8	31.9 ± 4.5	27.8 ± 2.4	28.3 ± 5.7	25.3 ± 1.3	24.3 ± 1.5
QRS (ms)	14.7 ± 4.1	13.1 ± 3.0	11.9 ± 1.9	10.4 ± 1.2	10.3 ± 1.2	9.8 ± 1.1	8.9 ± 0.8
QTc (ms)	58.0 ± 5.6	54.5 ± 4.7	51.2 ± 4.5	45.5 ± 3.7	45.8 ± 4.7	43.8 ± 2.7	42.6 ± 2.1
pNN6 (ms)	0.4 ± 1.2	3.0 ± 11.7	16.3 ± 30.0	8.0 ± 17.2	13.7 ± 23.1	7.3 ± 11.4	3.1 ± 7.2
							

Heart rate (HR), heart rate variability (HRVar), PR interval, QRS interval, corrected QT interval (QTc), and percentage of adjacent intervals differing by greater than 6 ms (pNN6) are provided. Values are expressed as mean ± SD.

To gain insight into the consistency of ECG recordings, a subset of 9 littermates were assayed 3-4 times each at all neonatal time points (data not shown). We found multiple recordings within individual mice to be highly consistent, varying by approximately ± 3% on average. Higher variation was observed in comparing the average recordings taken between littermates, which was comparable to the variation of mean values between different litters (both approximately ± 6%).

PR intervals rapidly decreased in duration from 37.5 ± 5.4 ms at PND4 to near adult levels of 24.3 ± 1.5 ms at PND14. The PR interval was precisely maintained, exhibiting consistent overlap between groups and low deviation throughout development. Representing time of ventricular depolarization, the QRS interval duration also decreased rapidly and was precisely maintained in the developing mouse. Conduction times reached their minimal value of 8.9 ± 0.8 ms by PND14, then levelled with a slight increase as the mouse aged. QT corrected for heart rate QT (QTc) showed similar developmental trends in the growing mouse, with minimal values of 42.6 ± 2.1 ms present by PND14.

### Heart rate variability in developing mice

**Figure 3:**
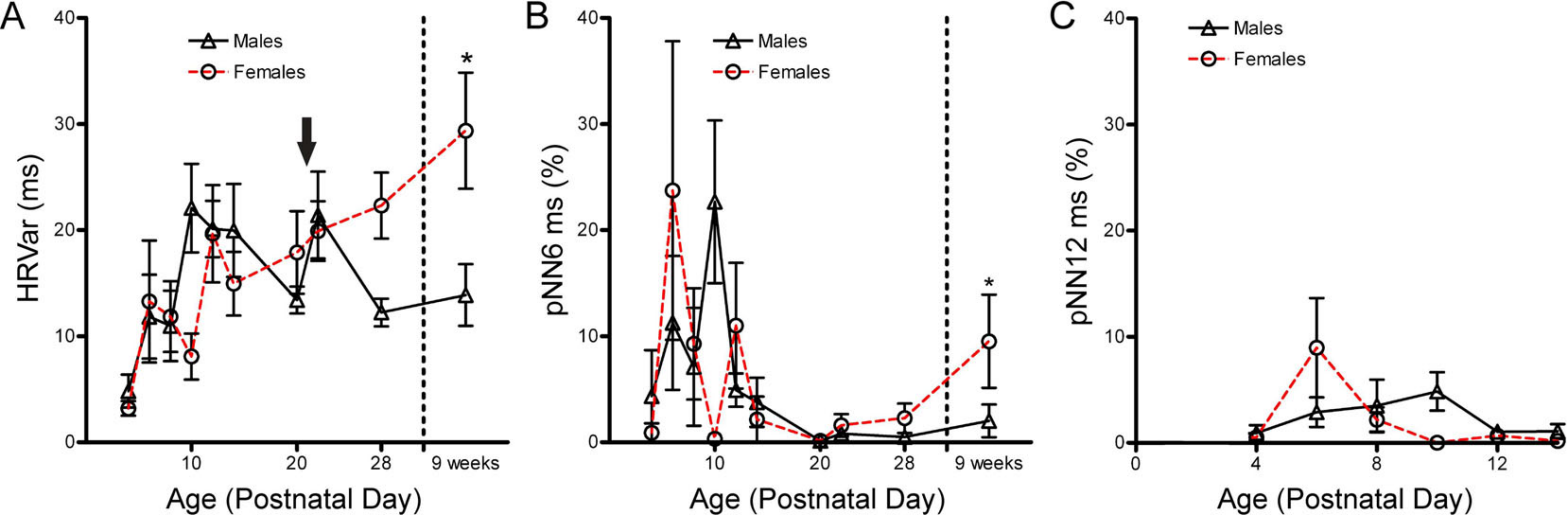
**Heart rate variability in developing mice**. A) Heart rate variability during development. (*P ≤ 0.05, linear mixed model). B) pNN6 throughout development (*P ≤ 0.05, logistic regression model). C) pNN12 displays the effect of altering the threshold in (B). Values are presented here as mean ± SEM. An interaction effect (*P *≤ 0.01) between sex and age is present in both A and B. Arrow denotes point at which mice were weaned.

We measured heart rate variability to query autonomic nervous system modulation of heart rhythm. We found heart rate variability to be lower in neonates than adults, initially being very low at PND2-4 before rising with age through 9 weeks (Figure 3A). A significant interaction effect (*P *≤ 0.01) between sex and age was detected over development. A consistent difference in heart rate variability was observed between sexes beginning at PND28. This effect was significant by adulthood, with females displaying higher variability than males (29.4 ± 15.5 ms versus 13.9 ± 10.1 ms, *P *≤ 0.05).

To examine a time domain statistic of heart rate variability, we determined the percentage of adjacent cardiac interbeat intervals that differed by greater than 6 ms (pNN6) for each of the different time points assayed (Figure 3B). In adult mice, this threshold value of 6 ms has been established as proportional to the value of 50 ms commonly used in human clinical studies [31,33]. We found all data points from PND14 onward displayed a value of less than 10%, with most less than 5%. These values are comparable to those found in adult humans in recent studies [34,35]. As with heart rate and heart rate variability, a strong interaction effect was present between gender and age (*P *≤ 0.01). A significant difference in pNN6 between mice of opposite sexes was observed at week 9, with adult females displaying a higher percentage of intervals exceeding the 6 ms threshold value than males (*P *≤ 0.05, logistical regression with repeated measurement). A difference was also observed at PND10, however this was not viewed as a true phenomenon due to the value here being near zero for females and this being inconsistent with pooled mice in a replication experiment (data not shown). Before PND14 higher percentages and an erratic pattern was observed. Note, this coincides with slower heart rates and thus larger RR intervals, as well as with some inconsistency in general heart rate variability. Together, these data suggest that pNNx may either require larger threshold values at younger neonate ages or there may be some instability in autonomic control of the heart during mid-neonatal ages. Addressing this first possibility, we re-analyzed the same ECG data using a larger threshold (12 ms). Values obtained for this pNN12 statistic in neonates were indeed more comparable to adult pNN6 values (Figure 3C).

## Discussion

We describe the development of resting heart rate, heart rate variability, and electrocardiographic parameters in conscious FVB/N mice, a commonly used research mouse strain, from day 2 of life to adulthood. We record ECG non-invasively without anaesthetic or restraint. Our methods potentiate similar studies in diverse disease models that are typically fragile and sensitive to anaesthetic or the stress associated with other methods of ECG recording, such as pin electrodes or surgical implantation of radio transmitters. The approach described and data reported should be useful to other investigators in designing and implementing studies that investigate the development of tachy- and brady-arrhythmias, heart block, conduction deficits, and modulation of heart rate by the autonomic nervous system in mouse models of human disease.

### Neonate ECG intervals

During neonatal development, we find heart rate increases linearly from PND2 through PND14. This high degree of homogeneity in heart rate data and linear temporal increase could facilitate identification of tachy- and brady-arrhythmias in mice with cardiac rhythm disturbances. Differences in slope or deviation from baseline could provide insight into the onset and progression of these arrhythmias.

Once reaching peak heart rates just at weaning, we observe a gradual decline in heart rate as animals age. Other ECG interval durations, such as PR interval, show slight prolongation with age as well. This is in agreement with previous aging studies in older groups of mice and together with the strong effects of age on ECG waveform intervals in developing mice (*P *≤ 0.0001) highlights the importance of age-matched control data [32].

The PR interval duration, reflecting time between atrial and ventricular depolarization, is an important indicator of heart block and cardiac conduction abnormalities. We show that PR intervals mature more quickly than heart rates do and are consistently and precisely regulated during development. This phenomenon could enable the sensitive detection of heart block throughout development, to add insight into the development of atrioventricular pathology in disease models.

The QRS interval duration (ventricular conduction) and rate corrected QT interval (repolarization) mature fairly rapidly and did not exhibit a significant sex difference at any point, though QTc did show a slight trend of higher values in females. These characteristics could enable detection of metabolic or respiratory deficiencies and possible drug toxicity independent of gender at any time point via alterations in ventricular conduction or repolarization.

### Onset of gender differences

Gender differences in heart rate and the ECG in FVB/N mice do not appear until weaning. Neonate mice may therefore be reliably and repeatedly assayed for ECG abnormalities without having to separate groups by sex. Upon weaning, females quickly develop a slower average heart rate than males, establishing a strong interaction effect between sex and age over development of mice. This does not appear to be caused by elongation of PR interval because no interaction effect or significant elongation is present at this time. Possible explanations for slower heart rate include a discrepancy in stress response to being separated from their dam, adjustment to an entirely dry-food diet, or hormonal changes taking place as mice approach sexual maturity [36].

Eventually, females develop elongated PR intervals. As in previous reports, we detect this elongation of PR interval relative to males at 9 weeks of age [30,31]. However, we find precisely maintained PR intervals with no sex difference present through PND28. This suggests developmental changes to cardiac conduction are still taking place between the ages of 4 and 9 weeks, and that the differentiation of heart rate and PR represent separate developmental events. Possible physiological roots for these differences include further myocardial changes, which are observed in continued atrioventricular valve and extracellular matrix development at 5 weeks of age [37], or hormonal shifts as mice reach sexual maturity manifesting in differential autonomic regulation [36].

### Measures of heart rate variability

Measures of variability in heart rate have demonstrated utility as indicators of impaired autonomic function and as signals predictive of adverse cardiac events [35,38-40]. We find heart rate variability to be lower in neonates than in adult mice, with values in young neonates (PND2-4) particularly low. This has been previously noted and found to be consistent in larger mammalian models and humans as well [41-44]. Variability may prove to be a useful parameter in studies of models associated with SIDS, in which a majority of sudden death can occur by PND5.

The pNN6 time domain statistic appears to be stable and useful from approximately PND12 onward. Before this, naturally larger RR intervals may necessitate the use of a threshold value of greater than 6 ms in order to obtain physiologically relevant insight in the neonate mouse. Results obtained for pNN12 measurements support this. Alternatively, there may be some instability in autonomic control of the heart at some neonate ages that could interfere with consistency in variability parameters. Supporting this alternative explanation, data from neonate rats indicates that autonomic neurotransmission continues to develop in the heart from PND4-15 [45], and that pharmacological modulation of the autonomic nervous system has altered/reduced effects in early neonates [46]. In adult mice, we observed pNN6 values to be consistent with control groups of humans in clinical studies [34,35]. Together, these demonstrate that variability parameters can be applied to mice of varying ages in studies of disorders such as sudden infant death syndrome (SIDS), sudden cardiac arrest, atrial fibrillation, aneurysm, congestive heart failure and sustained ventricular tachycardia.

### Utility and future study design

Resting heart rate, PR interval, QRS and heart rate variability can be assayed consistently through a non-invasive method in neonate mice as early as PND2. During neonatal stages we are often but not always able to resolve developmental differences in ECG parameters at two day intervals. For efficiency of experiments, surveys of four-day intervals showed highly significant differences and may be preferable in detecting changes over time where mouse models and experimental design allow. Practically, we found that an acclimation period was necessary to obtain stable readings. Once they acclimate, individual mice display consistent values within each time point. Variation between littermates was higher than within individuals and was observed to be comparable to that between separate litters. With rapid developmental changes and slow elongation of several parameters during aging at adult time points, we recommend including age-matched littermate controls whenever possible. Sex-matching of sample groups appears to be unnecessary in studies focusing either on pre-weaned mice or on parameters such as QRS and QTc, which never display a difference between males and females.

## Conclusions

Despite widespread clinical use of ECG in monitoring arrhythmias, little information exists on the establishment of ECG patterns and abnormalities in mouse models of disease. We have used a non-invasive system to characterize the development of ECG patterns in conscious FVB/N neonate mice. We confirm sex-based differences in heart rate and PR interval of adult mice while establishing when they differentiate, at two different points in development. Trends found here demonstrate the utility of examined parameters in various postnatal stages of mouse development and establish baseline data against which to detect the presence of arrhythmias of varying types and indications. Together, this information should be of broad utility in the design and implementation of studies in a wide variety of human disease models.

## Methods

### Animal maintenance

Wild type FVB/N mice were purchased from The Jackson Laboratories. All animals were maintained in a controlled animal facility at 20°C and 50% humidity, with a photoperiod of 12 h light/12 h dark where they were monitored daily for health as described previously [47]. Mice were fed *ad libitum *for water and food on a Harlan 6% crude fat diet. All maintenance and procedures were approved and performed in accordance with the Children's Memorial Research Center's Institutional Animal Care and Use Committee regulations.

### Electrocardiography

ECGs were recorded non-invasively in conscious mice using the ECGenie system (Mouse Specifics) as detailed previously by Chu et al [30]. Recordings were initially performed every two days from PND4 through PND14. After initial assays, we found consistent readings were obtainable as early as PND2. Neonate mice were placed inside of a temperature-controlled heated cup positioned on the ECG platform set to maintain a temperature of 30°C. Body temperatures were measured on a subset of mice using an infrared thermometer to verify consistency before and after taking ECG readings. For weanling and adult time points, readings were obtained on the days before (PND20) and after (PND22) weaning, as well as at PND28 and week nine. Briefly, mice were placed on a small platform instrumented with ECG recording electrodes. All mice were allowed to acclimate for ~10 minutes. After the acclimation period, ECG signals were recorded for ~5 seconds while the mice passively established contact between the underside of their paws and the electrodes.

Data acquisition was carried out longitudinally over development using the program LabChart 6 (ADInstruments). Analysis of individual ECG signals was then performed using e-MOUSE physiologic waveform analysis software (Mouse Specifics, Inc.) [30]. In this system, ECG recordings were filtered/assessed by the user before being analyzed by automated algorithms. Signals which were determined to contain too much noise or incorrectly called waveforms could be trimmed or removed and replaced with another reading taken from the same mouse on the same day. Once trained and when screening data quality simultaneously to data acquisition, users were capable of successfully acquiring usable data from all mice barring outside events that interfered with the resting physiological state of mice. Readings obtained from mice noted to experience a trauma which may interfere with resting heart rate (i.e. death, cage flooding, bleeding/bite marks from fighting) were excluded from analysis or absent from acquisition. Readings for all time points from such mice were removed from the experiments here for both presentation of data and statistical analyses, and used instead to provide a mixed pool reference to support findings from the primary longitudinal experiment. In total, at least thirty mice were assayed for each time point. All data was obtained during daylight hours, at which point the mouse heart rate is stable before increasing during the more active evening/night hours.

In evaluating waveforms and intervals, the end of the T wave was determined as the return of the signal to the isoelectric line as previously described [30]. QTc was calculated according to Bazett's formula modified specifically for mice, as reported by Mitchell et al [28]: QTc = QT_0 _/[(RR_0_/100)^1/2^]. HRVar represents the standard deviation of RR intervals. pNNx values are the percentage of adjacent RR intervals that differ in duration by greater than the threshold value of "x" ms. For example, pNN6 is defined as the percentage of adjacent RR intervals that display a difference exceeding 6 ms, where the threshold 6 ms was chosen because pNN6 has been previously identified in mice to correlate to the clinically important value of pNN50 [33].

### Statistical analyses

Data are presented as mean ± SD unless otherwise noted. All statistical analyses were performed on twenty mice chosen randomly at birth (8 females, 12 males) and assayed longitudinally at all time points. A linear mixed model with the random intercept was applied to analyze the relationship of gender and age effects on heart rate, PR interval, QRS interval, QTc interval, and heart rate variability throughout development. A logistic regression model with repeated measurement was applied to analyze pNN6 at different ages between male and female groups. P-values for pair-wise comparisons were adjusted using the Bonferronni method. The analysis was conducted using SAS 9.2. Differences were considered significant when *P *≤ 0.05.

## Authors' contributions

CRH carried out the animal work, collected ECG recordings, performed the analyses, designed the study and drafted the manuscript. TGH provided training on how to perform ECG and intellectual insight into cardiology, study design and electrocardiography. DW performed the statistical analyses, results interpretation and assisted with manuscript revision. CJD participated in conception and design of the study, provided funding for the work, and helped draft the manuscript. All authors read and approved the final manuscript.
